# Supplementary material for: Modeling glioblastoma heterogeneity as a dynamic network of cell states
Source: Mol Syst Biol. 2021 Sep 16;17(9):e10105. doi: 10.15252/msb.202010105 (PMC8444284; doi:10.15252/msb.202010105)
Supplement: Supplementary file 5 — Source Data for Figure 3 [file MSB-17-e10105-s001.zip › Figure3A_sourcedata/GSEA_3065/hallmarks_state4.GseaPreranked.1623416296068/HALLMARK_ESTROGEN_RESPONSE_LATE.html]

Details for gene set HALLMARK\_ESTROGEN\_RESPONSE\_LATE[GSEA]

|  || Dataset | state4 |
| Phenotype | NoPhenotypeAvailable |
| Upregulated in class | na\_neg |
| GeneSet | HALLMARK\_ESTROGEN\_RESPONSE\_LATE |
| Enrichment Score (ES) | -0.42885515 |
| Normalized Enrichment Score (NES) | -1.5094477 |
| Nominal p-value | 0.010810811 |
| FDR q-value | 0.048979837 |
| FWER p-Value | 0.498 |
Table: GSEA Results Summary

  

Fig 1: Enrichment plot: HALLMARK\_ESTROGEN\_RESPONSE\_LATE      
 Profile of the Running ES Score & Positions of GeneSet Members on the Rank Ordered List

  

| PROBE | GENE SYMBOL | GENE\_TITLE | RANK IN GENE LIST | RANK METRIC SCORE | RUNNING ES | CORE ENRICHMENT || 1 | RAB31 |  |  | 21 | 0.388 | 0.0323 | No |
| 2 | FABP5 |  |  | 23 | 0.375 | 0.0668 | No |
| 3 | ID2 |  |  | 56 | 0.292 | 0.0885 | No |
| 4 | CHPT1 |  |  | 155 | 0.220 | 0.0929 | No |
| 5 | COX6C |  |  | 269 | 0.181 | 0.0913 | No |
| 6 | ETFB |  |  | 315 | 0.172 | 0.0998 | No |
| 7 | ALDH3A2 |  |  | 578 | 0.128 | 0.0692 | No |
| 8 | IDH2 |  |  | 615 | 0.124 | 0.0748 | No |
| 9 | ARL3 |  |  | 657 | 0.119 | 0.0792 | No |
| 10 | PDCD4 |  |  | 796 | 0.104 | 0.0664 | No |
| 11 | MOCS2 |  |  | 828 | 0.102 | 0.0708 | No |
| 12 | FOS |  |  | 832 | 0.101 | 0.0796 | No |
| 13 | BLVRB |  |  | 841 | 0.100 | 0.0876 | No |
| 14 | SNX10 |  |  | 1002 | 0.088 | 0.0698 | No |
| 15 | CA2 |  |  | 1037 | 0.085 | 0.0722 | No |
| 16 | MAPT |  |  | 1065 | 0.084 | 0.0755 | No |
| 17 | PRKAR2B |  |  | 1084 | 0.083 | 0.0803 | No |
| 18 | ADD3 |  |  | 1118 | 0.081 | 0.0824 | No |
| 19 | CA12 |  |  | 1205 | 0.076 | 0.0755 | No |
| 20 | FAM102A |  |  | 1221 | 0.075 | 0.0799 | No |
| 21 | TOB1 |  |  | 1439 | 0.065 | 0.0508 | No |
| 22 | UNC13B |  |  | 1746 | 0.054 | 0.0062 | No |
| 23 | CKB |  |  | 1774 | 0.053 | 0.0067 | No |
| 24 | RPS6KA2 |  |  | 1830 | 0.051 | 0.0026 | No |
| 25 | PDLIM3 |  |  | 2070 | 0.045 | -0.0320 | No |
| 26 | TST |  |  | 2118 | 0.043 | -0.0356 | No |
| 27 | JAK1 |  |  | 2187 | 0.042 | -0.0427 | No |
| 28 | DLG5 |  |  | 2642 | 0.032 | -0.1132 | No |
| 29 | CD44 |  |  | 2687 | 0.031 | -0.1175 | No |
| 30 | FGFR3 |  |  | 2779 | 0.029 | -0.1295 | No |
| 31 | SLC26A2 |  |  | 2805 | 0.028 | -0.1310 | No |
| 32 | RABEP1 |  |  | 2863 | 0.027 | -0.1377 | No |
| 33 | IL6ST |  |  | 3218 | 0.021 | -0.1931 | No |
| 34 | ABCA3 |  |  | 3335 | 0.017 | -0.2103 | No |
| 35 | AFF1 |  |  | 3478 | 0.013 | -0.2321 | No |
| 36 | DYNLT3 |  |  | 3511 | 0.012 | -0.2362 | No |
| 37 | PLXNB1 |  |  | 3582 | 0.009 | -0.2466 | No |
| 38 | FARP1 |  |  | 3631 | 0.008 | -0.2537 | No |
| 39 | ITPK1 |  |  | 3750 | 0.003 | -0.2724 | No |
| 40 | AMFR |  |  | 3801 | 0.001 | -0.2805 | No |
| 41 | CELSR2 |  |  | 3946 | -0.007 | -0.3032 | No |
| 42 | SEMA3B |  |  | 3975 | -0.008 | -0.3070 | No |
| 43 | FDFT1 |  |  | 4088 | -0.014 | -0.3238 | No |
| 44 | DCXR |  |  | 4188 | -0.021 | -0.3378 | No |
| 45 | ATP2B4 |  |  | 4292 | -0.028 | -0.3519 | No |
| 46 | ISG20 |  |  | 4361 | -0.033 | -0.3599 | No |
| 47 | SLC29A1 |  |  | 4690 | -0.059 | -0.4075 | No |
| 48 | MEST |  |  | 4739 | -0.063 | -0.4095 | No |
| 49 | GALE |  |  | 4750 | -0.064 | -0.4053 | No |
| 50 | TFAP2C |  |  | 4780 | -0.066 | -0.4038 | No |
| 51 | TSPAN13 |  |  | 4822 | -0.069 | -0.4041 | No |
| 52 | TPBG |  |  | 4976 | -0.081 | -0.4214 | Yes |
| 53 | CPE |  |  | 5002 | -0.083 | -0.4178 | Yes |
| 54 | FKBP5 |  |  | 5047 | -0.087 | -0.4169 | Yes |
| 55 | CXCL12 |  |  | 5079 | -0.089 | -0.4137 | Yes |
| 56 | MDK |  |  | 5110 | -0.093 | -0.4100 | Yes |
| 57 | HOMER2 |  |  | 5194 | -0.101 | -0.4142 | Yes |
| 58 | PLK4 |  |  | 5210 | -0.102 | -0.4072 | Yes |
| 59 | ELOVL5 |  |  | 5226 | -0.103 | -0.4001 | Yes |
| 60 | RNASEH2A |  |  | 5241 | -0.105 | -0.3927 | Yes |
| 61 | ABHD2 |  |  | 5255 | -0.107 | -0.3850 | Yes |
| 62 | PRSS23 |  |  | 5258 | -0.107 | -0.3755 | Yes |
| 63 | KIF20A |  |  | 5260 | -0.107 | -0.3658 | Yes |
| 64 | PPIF |  |  | 5337 | -0.112 | -0.3677 | Yes |
| 65 | XBP1 |  |  | 5441 | -0.123 | -0.3731 | Yes |
| 66 | FKBP4 |  |  | 5480 | -0.129 | -0.3673 | Yes |
| 67 | SLC7A5 |  |  | 5488 | -0.131 | -0.3564 | Yes |
| 68 | HPRT1 |  |  | 5554 | -0.136 | -0.3543 | Yes |
| 69 | GLA |  |  | 5694 | -0.159 | -0.3622 | Yes |
| 70 | DNAJC1 |  |  | 5696 | -0.160 | -0.3476 | Yes |
| 71 | SLC16A1 |  |  | 5794 | -0.182 | -0.3466 | Yes |
| 72 | CAV1 |  |  | 5842 | -0.193 | -0.3363 | Yes |
| 73 | RBBP8 |  |  | 5854 | -0.195 | -0.3201 | Yes |
| 74 | PERP |  |  | 5859 | -0.196 | -0.3026 | Yes |
| 75 | BTG3 |  |  | 5895 | -0.206 | -0.2894 | Yes |
| 76 | EMP2 |  |  | 6032 | -0.254 | -0.2879 | Yes |
| 77 | DHCR7 |  |  | 6083 | -0.287 | -0.2696 | Yes |
| 78 | CDC6 |  |  | 6103 | -0.299 | -0.2451 | Yes |
| 79 | CDC20 |  |  | 6154 | -0.344 | -0.2214 | Yes |
| 80 | CD9 |  |  | 6176 | -0.378 | -0.1900 | Yes |
| 81 | GINS2 |  |  | 6194 | -0.420 | -0.1540 | Yes |
| 82 | CCND1 |  |  | 6203 | -0.454 | -0.1135 | Yes |
| 83 | TFPI2 |  |  | 6230 | -0.518 | -0.0699 | Yes |
| 84 | TOP2A |  |  | 6260 | -0.814 | 0.0005 | Yes |
Table: GSEA details [plain text format]

  

Fig 2: HALLMARK\_ESTROGEN\_RESPONSE\_LATE: Random ES distribution      
 Gene set null distribution of ES for **HALLMARK\_ESTROGEN\_RESPONSE\_LATE**
